# An Update on Predictive Biomarkers for Treatment Selection in Non-Small Cell Lung Cancer

**DOI:** 10.3390/jcm7060153

**Published:** 2018-06-15

**Authors:** Tamkin Ahmadzada, Steven Kao, Glen Reid, Michael Boyer, Annabelle Mahar, Wendy A. Cooper

**Affiliations:** 1 Sydney Medical School, The University of Sydney, Sydney 2006, Australia; steven.kao@lh.org.au (S.K.); glen.reid@sydney.edu.au (G.R.); michael.boyer@lh.org.au (M.B.); wendy.cooper@health.nsw.gov.au (W.A.C.); 2 Chris O’Brien Lifehouse, Sydney 2050, Australia; 3 Asbestos Diseases Research Institute (ADRI), Sydney 2139, Australia; 4 Tissue Pathology and Diagnostic Oncology, Royal Prince Alfred Hospital, Sydney 2050, Australia; annabelle.mahar@health.nsw.gov.au; 5 School of Medicine, Western Sydney University, Sydney 2560, Australia

**Keywords:** NSCLC, predictive biomarkers, targeted therapy, immunotherapy

## Abstract

It is now widely established that management of lung cancer is much more complex and cannot be centered on the binary classification of small-cell versus non-small cell lung cancer (NSCLC). Lung cancer is now recognized as a highly heterogeneous disease that develops from genetic mutations and gene expression patterns, which initiate uncontrolled cellular growth, proliferation and progression, as well as immune evasion. Accurate biomarker assessment to determine the mutational status of driver mutations such as *EGFR*, *ALK* and *ROS1*, which can be targeted by specific tyrosine kinase inhibitors, is now essential for treatment decision making in advanced stage NSCLC and has shifted the treatment paradigm of NSCLC to more individualized therapy. Rapid advancements in immunotherapeutic approaches to NSCLC treatment have been paralleled by development of a range of potential predictive biomarkers that can enrich for patient response, including PD-L1 expression and tumor mutational burden. Here, we review the key biomarkers that help predict response to treatment options in NSCLC patients.

## 1. Introduction

For several decades, lung cancer has been the leading cause of cancer-related deaths worldwide [1]. According to the latest GLOBOCAN database, lung cancer accounted for around 19% of all cancer-related deaths worldwide in 2012 [2]. Prognosis is poor in lung cancer patients, with over 50% dying within one year following diagnosis and a five-year survival rate of less than 20% [1,3]. There is an urgent need to improve clinical management of lung cancer patients and biomarkers can assist in selecting optimal treatment regimes.

Traditionally, lung cancer has been categorised into two main sub-types: small cell lung cancer (SCLC) and non-small cell lung cancer (NSCLC). SCLC accounts for the 15% of all lung cancer cases. NSCLC is more common, accounting for the remaining 85% of cases and will be the focus of this review, as currently biomarkers predominantly play a role in this setting. Approximately two thirds of NSCLC patients are diagnosed late with advanced or metastatic disease (stage III/IV) [4,5] and by this stage, treatment options are limited, prognosis is poor and survival rates are low. For this reason, there is a strong unmet clinical need for new biomarkers that can help with early diagnosis, improve prognostication and predict response to various therapies, enabling more individualised patient treatment.

A biomarker is any characteristic that can be measured and that gives an indication of the biological state of the patient or their tumor. Diagnostic biomarkers can differentiate the disease of interest from other diseases or normal state; prognostic biomarkers provide information about disease outcome and the pace of progression regardless of treatment; and predictive biomarkers indicate whether a particular treatment is likely to provide a clinical benefit for a patient. Some biomarkers may be more appropriately defined as theranostic markers, which have the ability to indicate targeted therapy based on a specific diagnostic test, however the term is currently not widely used in clinical practice. An ideal biomarker should be inexpensive, reproducible, easy to obtain and easily sampled with a minimally invasive technique [6]. While diagnostic and prognostic biomarkers can help improve management of lung cancer patients, it could be argued that predictive biomarkers are the most important as they have the potential to personalise treatments for patients [7]. Predictive biomarkers can potentially reduce the costs and toxicities associated with ineffective treatments, particularly for patients who are candidates of targeted therapies [8]. Here, we aim to review predictive biomarkers that are currently used in clinical practice for treatment selection in NSCLC patients. We also review predictive biomarkers that have potential to be translated into clinical practice in the near future.

## 2. Biomarkers Used in NSCLC

### 2.1. Predictive Factors for Chemotherapy in NSCLC

Given that the majority of NSCLC patients are diagnosed at late stages of the disease, chemotherapy has been the only approved systemic therapy for NSCLC patients for several years. However, chemotherapy provides only modest benefit (median survival of 10 months) with substantial risk of toxicity [7,9,10]. There are currently no clinically validated predictive biomarkers that can customize the choice of chemotherapy drugs for NSCLC patients [11]. Histologic subtype of NSCLC is currently the only predictive factor used in clinical practice to guide chemotherapy treatment. Nonsquamous histology has been shown to be a predictive factor in the use of pemetrexed chemotherapy [12]. Several clinical trials have demonstrated that pemetrexed, when given as a single agent or combined with a platinum analog, provided survival benefit in patients with nonsquamous NSCLC, but showed only some activity in squamous cell patients [7,13,14]. For advanced stage squamous cell NSCLC patients, platinum combined with paclitaxel, docetaxel, gemcitabine or vinorelbine are currently the standard treatment options [15]. Other predictive biomarkers for chemotherapy have been investigated in preclinical studies, including expression of excision repair cross-complement group 1 (*ERCC1*) enzyme, thymidylate synthase (TYMS), ribonucleotide reductase regulatory subunit M1 (*RRM1*) and breast cancer-specific tumor suppressor protein 1 (*BRCA1*) for platinum-based chemotherapy, however they have not yet demonstrated predictive utility sufficient for routine clinical practice [7].

### 2.2. Predictive Biomarkers for Targeted Therapies in NSCLC

NSCLC is a highly heterogeneous disease that develops from genetic mutations and gene expression patterns, which initiate uncontrolled cellular growth, proliferation and progression. Better understanding of the molecular biology of NSCLC has enabled targeted therapies to be developed for more personalised treatment. There are two main types of targeted therapies currently available for NSCLC patients: tyrosine kinase inhibitors (TKIs) [7,16] and monoclonal antibodies, which are primarily used in immunotherapy. Tyrosine kinases are enzymes that play an important role in cellular processes such as cell signalling, proliferation and differentiation [16]. Receptor tyrosine kinases are glycoproteins expressed on cell membranes that are activated when they bind to a specific ligand. These enzymes can become constitutively activated in cancer cells, leading to abnormal growth. TKIs target specific genetic mutations and block receptor activations to prevent signalling pathways and inhibit further growth of cancer cells.

The most important driver mutations found in NSCLC are *epidermal growth factor receptor* (*EGFR*), *kirsten rat sarcoma virus* (*KRAS*), *B-Raf* proto-oncogene (*BRAF*) and *ERBB2* (*HER2*) mutations, rearrangements in the *anaplastic lymphoma kinase* (*ALK*), *c-ros* oncogene 1 (*ROS1*), and *RET* genes, and *MET* amplifications [17,18]. Of these, sensitizing *EGFR* mutations, *ALK* and *ROS1* rearrangements as well as *BRAF* mutations have targeted therapies approved by the US Food and Drug Administration (FDA) in NSCLC (Table 1).

### 2.3. Epidermal Growth Factor Receptor Mutations in NSCLC

EGFR is a growth factor receptor expressed on the surface of cells that is involved in cell growth and division following ligand binding [7]. However, activating *EGFR* mutations can result in constitutive activation of the receptor, independent of ligand binding, leading to tumor development and growth. *EGFR* mutations are more commonly observed in patients with light or no smoking history, female patients, adenocarcinomas or NSCLC with an adenocarcinoma component [4,19,20]. Inhibition of mutated EGFR can be achieved with targeted TKIs [4], which work by selectively blocking phosphorylation of the intracellular tyrosine kinase domain of EGFR [19]. Most *EGFR* mutations occur in exons 18 to 21 of the tyrosine kinase domain [19], with the most common mutations being exon 19 deletions and a point mutation in exon 21 (L858R) [7,16]. These two mutations are known as activating or sensitizing *EGFR* mutations because they result in sensitivity to TKIs. They have been observed in approximately 15% of lung adenocarcinomas in Western populations and 25–50% in Asian populations [19]. Current FDA-approved TKIs for sensitizing *EGFR* mutations include erlotinib, gefitinib, osimertinib and afatinib [7,16,21]. Data from several trials have shown that sensitizing *EGFR* mutations can predict a response rate to TKIs of 65–90% in advanced NSCLC patients and an overall survival of approximately 24 months [19,22]. By contrast, patients with tumours that are wild-type for *EGFR* have better outcomes with conventional platinum-based chemotherapy than EGFR TKIs and lack of an activating *EGFR* mutation could be considered a contraindication to EGFR TKI therapy [23]. Other rare *EGFR* mutations include substitutions such as glycine 719 with serine, cysteine or alanine in exon 18, which confer sensitivity to EGFR TKIs, or mutations associated with resistance to first generation TKIs such as the T790M mutation in exon 20 or insertions in exon 20 [19,21]. Preliminary evidence from the LUX-Lung 2, LUX-Lung 3, and the LUX-Lung 6 trials suggest that afatinib may also be active in some of these rarer *EGFR* mutations [24].

Current evidence-based consensus guidelines on molecular testing in lung cancer patients from the College of American Pathologists, International Association of Lung Cancer and the Association for Molecular Pathologists, recommend that all patients with advanced stage lung adenocarcinoma (or with an adenocarcinoma component), regardless of clinical features, should undergo biomarker testing for *EGFR* mutations and *ALK* and *ROS1* rearrangements [25]. Histological or cytological samples may be used for testing if there is adequate tumor cellularity, and primary or metastatic sites are appropriate for biomarker assessment. While a variety of mutation testing modalities can be used to detect *EGFR* mutations, it is recommended that assays are sensitive enough to detect alterations in formalin-fixed paraffin-embedded (FFPE) specimens with tumor cell content as low as 20% [25], and that all activating *EGFR* mutations in exons 18–21 with a prevalence of at least 1% are covered by the assay. Although mutation-specific immunohistochemical stains for the L858R mutation and a subset of exon 19 deletions have demonstrated accuracy [18], they are suboptimal for detection of all relevant *EGFR* mutations in the clinical setting and are not recommended for routine use [25].

Patients treated with EGFR TKIs develop drug resistance over time through a variety of mechanisms including secondary acquired mutations that render the TKIs ineffective. The most common resistance mutation to first generation TKIs is the T790M mutation in exon 20 (in which threonine becomes replaced by methionine at position 790 in the tyrosine kinase domain of *EGFR*) [7], which reduces the effectiveness of early generation EGFR TKIs. Osimertinib is currently approved for advanced NSCLC patients with T790M mutation, with disease progression on or after EGFR TKI therapy [16] and is recommended as a subsequent therapy (previously referred to as second-line therapy) for patients with metastatic *EGFR* T790M-positive NSCLC [15]. Biomarker testing for *EGFR* T790M mutation is therefore recommended for *EGFR* mutant patients that have progressed following EGFR TKI treatment. Assays with high analytic sensitivity are recommended in this setting [25]. However, this approach will not necessarily ensure that all relevant mutations are identified as tumour heterogeneity may not be sufficiently represented in biopsies from a single site. Presence of potentially pre-existing undetected mutant subpopulations that could drive therapeutic resistance may be missed [26]. Data from clinical trials have suggested allele-specific real-time polymerase chain reaction assays that would be capable of detecting *EGFR* T790M mutations with as few as 5% tumor cells. In clinical practice, careful assay validation is warranted in order to establish appropriate sensitivity of the method being used. Detection of the T790M mutation in circulating tumor DNA from plasma samples (“liquid biopsies”) has been suggested as it has the advantage of potentially identifying mutations from multiple disease sites that could harbour different tumour subclones. However, testing on a tumor sample is recommended if the plasma sample is negative due to the low negative predictive value and low sensitivity of the technique [25].

### 2.4. Anaplastic Lymphoma Kinase Rearrangements in NSCLC

The *ALK* gene encodes for a receptor tyrosine kinase that is thought to transmit growth activating signals [27]. Activating *ALK* gene rearrangements that produce an abnormally, constitutively expressed and activated ALK protein led to abnormal cell growth and proliferation. *ALK* rearrangements are found in approximately 2–7% of patients with NSCLC, almost all of which are adenocarcinomas [7,16,28]. The most common rearrangement occurs when the *ALK* gene fuses with the echinoderm microtubule-associated protein like 4 (*EML4*) gene through an inversion, producing the *EML4-ALK* fusion oncogene. *ALK* rearrangement is more commonly found in younger patients with adenocarcinoma histology who are light smokers or who have never smoked, and they are almost always mutually exclusive with other oncogenic drivers such as *EGFR* mutations [16,20,27].

Biomarkers to detect *ALK* rearranged NSCLC include immunohistochemistry (IHC) [29] and fluorescence in situ hybridization (FISH) using a break apart assay [25,27,28,29,30]. Molecular methods such as reverse transcriptase-polymerase chain reaction (RT-PCR) or next generation sequencing (NGS) can also be utilized but unusual or novel fusion partners or isoforms are not detected by RT-PCR or amplicon-based NGS techniques. In the USA, IHC is approved as an alternative to FISH for ALK biomarker assessment, although in other countries, IHC is used for screening with FISH used as a confirmatory technique.

Biomarker detection of *ALK* rearranged NSCLC is essential as these patients are best treated with targeted ALK TKIs such as crizotinib, showing objective response rates of 74% compared to 45% with conventional chemotherapy [31]. Similar to EGFR targeted therapy, *ALK* positive patients can develop resistance to targeted TKIs. Newer generations of *ALK* inhibitors have been developed for patients who develop drug resistance or who cannot tolerate crizotinib—such as ceritinib [32], alectinib, brigatinib [16,33] and lorlatinib [32]. While molecular testing can be undertaken to identify various acquired resistance mutations in *ALK*, this is not currently routine clinical practice.

### 2.5. ROS1 Rearrangements in NSCLC

ROS1 is a receptor tyrosine kinase that plays a key role in cell growth and differentiation. *ROS1* rearrangements constitute a small subset of advanced NSCLC patients (1–3% of lung adenocarcinomas) [34] and are more common in light smokers or never smokers. Although ROS1 is a distinct receptor tyrosine kinase, it is structurally similar to the ALK protein due to similarities in their kinase domains and ATP binding sites [15,35]. The patient and disease characteristics of NSCLC patients with *ALK* alterations are also similar to NSCLC patients with *ROS1* gene alterations. *ROS1* rearranged NSCLCs can also be targeted by specific TKIs such as crizotinib [35,36]. To date, crizotinib is the only TKI that is FDA-approved for the treatment of *ROS1* positive advanced NSCLC [34]. Other targeted inhibitors are currently being investigated and are under development for *ROS1* rearranged NSCLC, including lorlatinib [32]; entrectinib [37], cabozantinib [38,39], ceritinib [33,40,41] and DS-6051b. Lorlatinib and carbozantinib are next-generation inhibitors for *ROS1* positive patients who have developed acquired resistance to crizotinib.

Biomarker testing for *ROS1* is recommended in all lung adenocarcinoma patients and can be performed using cytogenetic techniques such as FISH using a break apart probe, or molecular techniques including RT-PCR and NGS [25]. As with detection of *ALK* rearrangement, IHC is useful to screen for ROS1 expression but a molecular or cytogenetic technique such as FISH is currently required for confirmation of *ROS1* rearrangement [35].

### 2.6. BRAF Mutations in NSCLC

*BRAF* is a member of the mitogen-activated protein kinase (MAPK) pathway involved in regulating cellular functions such as proliferation, differentiation and apoptosis [20]. The MAPK pathway includes other signaling molecules such as *MEK*. Changes in the *BRAF* gene can produce altered BRAF proteins that promote abnormal cell growth. *BRAF* mutations occur in 2–5% of NSCLC and, like *EGFR*, are generally identified by molecular sequencing-based techniques [42]. The most common *BRAF* mutation observed is a glutamate substitution for valine at codon 600 (V600E), observed in 1–2% of NSCLC patients [20,43]. Unlike in melanoma, non-V600 mutations are relatively common in lung cancer accounting for 50–89% of *BRAF* mutations [42]. The clinical characteristics of NSCLC patients with *BRAF* mutations are not well defined, perhaps due to the low frequency observed in NSCLC patients, however they are strongly associated with adenocarcinoma histology [20]. Some studies have suggested that *BRAF*-mutant patients are more commonly former or current smokers, although there is limited evidence of smoking history for V600E-mutant patients [20].

Dabrafenib is a BRAF inhibitor that targets the *BRAF* V600E mutation. Another TKI, known as trametinib, is a MEK inhibitor that targets MEK proteins along the MAPK signaling pathway and can be used when patients develop acquired resistance to dabrafenib. Inhibition of the *BRAF* mutation, either alone or in combination with MEK inhibitors, has been successful in metastatic melanoma. This combination therapy has recently shown clinical efficacy in NSCLC patients. In 2017, the US Food and Drug Administration approved the BRAF TKI dabrafenib and the MEK inhibitor trametinib as a combination therapy for metastatic NSCLC patients with *BRAF* V600E mutation, following response rates of 61% in a phase 2 clinical trial [44]. Another BRAF V600E-mutant inhibitor, vemurafenib, is also being investigated for NSCLC [40].

### 2.7. Other Candidate Predictive Biomarkers

*KRAS* is a gene that encodes for rat sarcoma (RAS) protein, located at the inner surface of the plasma membrane and involved in the transduction of growth signals from RTKs [16]. *KRAS* mutations are common in Caucasians as well as current and former smokers [16] and are the most common activating mutation in NSCLC. The prevalence of *KRAS* mutations have been reported in 25% to 40% of lung adenocarcinomas (using standard clinical assays with an analytic sensitivity of at least 5%) [16,45]. *KRAS* mutations currently serve as prognostic biomarkers, as patients with *KRAS* mutations have shorter survival than those with wild-type *KRAS*. Targeting *RAS* mutations has been unsuccessful to date and there are currently no targeted therapies available for *KRAS* mutations [15,16]. As *KRAS* mutations are usually not detected together with *EGFR* mutations (using standard clinical assays), or *ALK* or *ROS1* rearrangements, the *KRAS* status can identify patients who are unlikely to benefit from further molecular testing and it can be a useful biomarker when part of a multigene panel [15,45].

Several candidate predictive biomarkers may also potentially be useful to identify patients for emerging targeted therapy approaches. These include *RET* rearrangements and *MET* alterations [14,16,20]. *MET* is considered a strong candidate given its response to crizotinib, currently a treatment for *ALK*- and *ROS1*-rearranged lung cancers [14,25], however, *MET* alterations are uncommon, occurring in approximately 5% of NSCLC. A range of *MET* alterations can lead to gene activation, making it a challenging biomarker to comprehensively assess. *MET* activation can occur through amplification [46], activating mutations in the tyrosine kinase domain, or a variety of complex exon 14 skipping mutations that prevent protein degradation [25]. The latter mutations are associated with sarcomatoid carcinomas, a rare histological subtype of NSCLC [47]. *ERBB2* (*HER2*) mutations or amplifications are also found in a small percentage of NSCLC (2–5%). *HER2* amplification has also been reported as a rare mechanism of resistance to EGFR inhibitors and could potentially be used as a biomarker for HER2 inhibitors, although there is currently insufficient evidence for routine clinical testing [25].

## 3. Multigene Panels for Molecular Biomarker Testing

Molecular biomarkers require sensitive, specific, and sufficiently fast and cost-effective tests for their determination. Ideally, a multigene (multiplexed) genetic sequencing panel covering hotspot mutations in a range of relevant genes such as in a next generation sequencing panel can be utilized to assess not only the essential predictive biomarkers (*EGFR*, *ALK* and *ROS1*) but also emerging targets such as *BRAF*, *MET*, *RET* and *ERBB2* (*HER2*). Next generation sequencing methods, either hybrid capture based, or amplicon based, have high sensitivity and are suitable for use on samples with low tumor cell concentration making them well suited for detection of mutations in the predominant clone of a tumour in clinical practice [25,48]. Any discrepant, unexpected or equivocal results should undergo testing using an alternative technique or an alternative sample to maximize the accuracy of biomarker assessment. 

## 4. Biomarkers for Immunotherapy

Immune evasion or immune resistance is a hallmark of cancer [49], in which cancer cells circumvent the immune system by several mechanisms including expressing immune suppressive receptors or by producing immunosuppressive proteins to inhibit the function of T lymphocytes. One of the mechanisms of immune resistance involves cancer cells using immune-inhibitory pathways called checkpoints. The two most widely studied checkpoints are the programmed death protein 1/programmed death receptor ligand 1 (PD-L1) and cytotoxic T-lymphocyte antigen 4 (CTLA-4) [9,16]. PD-1 is found to be more broadly expressed than CTLA-4 and can be expressed on T lymphocytes and non-T lymphocytes such as B cells and natural killer cells [9]. Currently, there is intense interest in the use of immunotherapy as a therapeutic option in cancer, especially NSCLC. Many tumors, including NSCLC, have increased expression of PD-L1 and utilise this as a mechanism of immune evasion. Therefore, PD-1/PD-L1 pathway blockade is a promising target in immunotherapy [9].

### 4.1. PD-L1 Expression as a Biomarker

PD-1 is expressed on effector lymphocytes, while its natural ligands, PD-L1 and to a lesser extent, PD-L2, are expressed on tumor cells or in the surrounding microenvironment. When PD-L1 binds to PD-1, it downregulates T cell function, inhibiting tumor rejection by the immune system. The PD-L1 checkpoints can be targeted by monoclonal antibodies that block PD-1/PD-L1 interaction [9]. There are currently four FDA-approved immunotherapy drugs targeting PD-L1 checkpoint in NSCLC: nivolumab, pembrolizumab, atezolizumab and durvalumab.

Pembrolizumab (a PD-1 inhibitor) is the first immunotherapy that was approved by the FDA for the treatment of patients with metastatic NSCLC, whose tumors express PD-L1 and requires confirmation of PD-L1 overexpression [16,50,51] (defined as expression in at least 50% of tumor cells), and no activating mutations of *EGFR* or *ALK* rearrangements in NSCLC patients with either squamous or nonsquamous histology. In the KEYNOTE-024 trial [50], the patients lived four months longer than patients who received standard platinum-based chemotherapy. High PD-L1 expression therefore has some power to predict response to pembrolizumab in first-line treatment in advanced NSCLC [9]. While the Dako PD-L1 22C3 pharmDx kit was developed as the predictive biomarker for pembrolizumab [52], recent studies have shown considerable (but not perfect) concordance with the Dako 28-8 and Ventana SP263 PD-L1 IHC clones [53,54,55]. Scoring of PD-L1 IHC using the 22C3 assay at the 50% cut-point can be achieved with 81.9% overall agreement between pathologists [56].

Currently, nivolumab, an IgG4 human moAB against PD-1, and atezolizumab a PD-L1 inhibitor, are FDA-approved for second line treatment of NSCLC without the need for a biomarker. While not essential for patient selection, PD-L1 IHC is considered a “complementary” biomarker for nivolumab that may assist in predicting response to treatment [57]. The PD-L1 inhibitor durvalumab is FDA-approved for patients with unresectable stage III NSCLC whose disease has not progressed following chemoradiotherapy, and biomarker selection of patients is not required [9].

### 4.2. Other Biomarkers for Immunotherapy

It is estimated that only 15–25% of NSCLC patients benefit from immunotherapy, suggesting there is a need to explore additional novel biomarkers [11,58]. In addition to PD-L1 expression, other biomarkers to predict response to immune checkpoint inhibitors in NSCLC have been examined including tumor mutational burden [59] and interferon-γ expression [60]. Most notably, NSCLC patients with a high tumor mutational burden (defined as ≥10 mutations per megabase using the FoundationOne CDx assay) treated with a combination of the immune checkpoint inhibitors nivolumab and ipilimumab (a CTLA-4 targeting antibody), showed greater progression-free survival than those treated with conventional chemotherapy in the first line setting [61]. However, there are currently many challenges with introducing tumor mutational burden testing into routine clinical practice including an accepted definition, cut-point to determine “high”, ability to reproduce results using different platforms/assays and difficulty obtaining biopsy specimens with enough quality DNA for assessment.

## 5. Challenges of Biomarker Assessment in NSCLC

Targeted therapies and immunotherapies require accurate biomarker assessment. One of the challenges is the low frequency of some targetable mutations in NSCLC, such as *ALK* and ROS1, and the need to test for a range of biomarkers. Overall, approximately 60% of tumors do not have any identifiable driver mutations [11]. There are also risks in obtaining sufficient patient biopsy samples, which often contain a low proportion of tumor cells and can be difficult to safely obtain in advanced NSCLC patients. Furthermore, at the time of drug acquired resistance, repeat biopsy is even more difficult and liquid biopsy approaches may be useful in this setting.

Plasma genotyping (also known as liquid biopsy or plasma biopsy) has the potential to overcome some of the challenges with pathological assessment in tissue biopsies, as it is minimally invasive, it can be repeated easily and can potentially provide a more comprehensive molecular profile of tumors at multiple metastatic sites. However, this approach currently lacks the required sensitivity with high positive predictive value but low negative predictive value, as there may be insufficient circulating free tumor DNA in plasma to identify any mutations [15,17].

## 6. Summary and Conclusions

NSCLC is a complex cancer and clinical management requires a good understanding of patient characteristics, tumor histology and comprehensive and accurate biomarker assessment. Discovery of targetable driver oncogenes, particularly *EGFR*, *ALK* and *ROS1*, along with the development of immunotherapeutic agents, have opened up new treatment paradigms for NSCLC with treatment decisions now relying on biomarker assessment. While new generation TKIs and immunotherapeutic approaches are rapidly evolving, not all patients benefit from their use, placing responsibility on the scientific and medical community to develop predictive biomarkers with the greatest clinical utility for optimal patient care. The heterogeneity of NSCLC remains a key barrier to accurate molecular classification and necessitates individualisation of treatment. Current biomarkers such as *EGFR*, *ALK*, *ROS1* and PD-L1 have led to improvements in the clinical management of NSCLC patients but there is still a need to better understand the mechanisms underlying efficacy of therapeutic agents and to investigate novel biomarkers to improve clinical management, response outcomes and cost-effectiveness of drugs in advanced NSCLC patients.

## Figures and Tables

**Table 1:** Predictive biomarkers for treatment selection in Non-Small Cell Lung Cancer.

Biomarker	Alteration of Interest	Assay	Frequency in NSCLC	FDA-Approved Targeted Therapies for NSCLC *
*EGFR*	Exon 19 deletion L858R point mutation in exon 21 L861Q point mutation in exon 21 G719X in exon 18 Other rarer activating mutations	PCR based mutation testing	~15% in Western populations ~35–50% in Asian populations	Erlotinib Afatinib Gefitinib Necitumumab
*EGFR*	T790M mutation in exon 20	PCR based mutation testing	60% in patients with disease progression following EGFR TKI	Osimertinib
*ALK*	*ALK* rearrangement	IHC ± FISH	3–7%	Crizotinib Ceritinib Alectinib Brigatinib
*ROS1*	*ROS1* rearrangement	IHC screening and FISH confirmation	1–2%	Crizotinib
*BRAF*	V600E mutation	PCR based mutation testing	1–3%	Dabrafenib Trametinib
PD-L1	High protein expression	IHC	~30% **	Pembrolizumab

* status of FDA approval current at the time of this review; ** Approximately 30% of advanced stage NSCLC have high PD-L1 expression (tumor proportion score of at least 50%).
